# Supplementary material for: Cross-cultural adaptation and validation of the Romanian International Knee Documentation Committee—subjective knee form
Source: PeerJ. 2020 Feb 3;8:e8448. doi: 10.7717/peerj.8448 (PMC7003694; doi:10.7717/peerj.8448)
Supplement: Supplemental Information 1 [file peerj-08-8448-s001.docx]

1. **IKDC CHESTIONAR DE EVALUARE A GENUNCHIULUI**

Constant

Niciodată

# 10

# □

# 9

# □

# 8

# □

# 7

# □

# 6

# □

# 5

# □

# 4

# □

# 3

# □

# 2

# □

# 0

# □

# 1

# □

**SIMPTOME*:**

*Evaluați simptomele la cel mai ridicat nivel de activitate pe care credeți că o puteți efectua fără probleme semnificative, chiar și dacă nu efectuați activități la acest nivel.

1. Care este cel mai ridicat nivel de activitate pe care îl puteți efectua fără dureri semnificative de genunchi?

- Activități fizice foarte intense, cum ar fi sărituri sau pivotari, ca în baschet sau fotbal
- Activități fizice intense, cum ar fi muncă fizică intensă, schi sau tenis
- Activități fizice moderate, cum ar fi muncă fizică moderată, alergare sau ,jogging’
- Activități fizice ușoare, cum ar fi mers pe jos, menaj sau lucru în curte
- Imposibilitatea de a efectua oricare dintre activitățile de mai sus din cauza durerii de genunchi

2. În ultimele 4 săptămâni, sau din momentul producerii leziunii, cât de des ați avut dureri?

Lună An

An

Zi

Zi

Lună

Data traumatismului: ______/ ______/ ______

Numele complet____________________________________________________________

Data de astăzi: ______/ ______/ ______

1. Dacă aveți durere, cât de severă este?

Fără durere

Durere cumplită

# 10

# □

# 9

# □

# 8

# □

# 7

# □

# 6

# □

# 5

# □

# 4

# □

# 3

# □

# 2

# □

# 1

# □

# 0

# □

1. În ultimele 4 săptămâni, sau din momentul producerii leziunii, cât de rigid sau umflat a fost genunchiul Dvs?

- Deloc
- Puțin
- Moderat
- Foarte mult
- Extrem

1. Care este cel mai înalt nivel de activitate pe care pe care îl puteți efectua fără ca genunchiul să se umfle semnificativ?

- Activități fizice foarte intense, cum ar fi sărituri sau pivotari, ca în baschet sau fotbal
- Activități fizice intense, cum ar fi muncă fizică intensă, schi sau tenis
- Activități fizice moderate, cum ar fi muncă fizică moderată, alergare sau ‚jogging’
- Activități fizice ușoare, cum ar fi mers pe jos, menaj sau lucru în curte
- Imposibilitatea de a efectua oricare dintre activitățile de mai sus din cauza umflării genunchiului

1. În ultimele 4 săptămâni, sau din momentul producerii leziunii, ați avut senzația de blocare a genunchiului?

**□**Da □Nu

1. Care este cel mai înalt nivel de activitate pe care îl puteți efectua fără să aveți senzația semnificativă de ‚genunchi care scapă’?
   - - Activități fizice foarte intense, cum ar fi sărituri sau pivotari, ca în baschet sau fotbal
     - Activități fizice intense, cum ar fi muncă fizică intensă, schi sau tenis
     - Activități fizice moderate, cum ar fi muncă fizică moderată, alergare sau ‚jogging’
     - Activități fizice ușoare, cum ar fi mers pe jos, menaj sau lucru în curte
     - Imposibilitatea de a efectua oricare dintre activitățile de mai sus din cauza senzației de ‚genunchi care scapă’

**ACTIVIT**ĂȚI SPORTIVE**:**

1. Care este cel mai înalt nivel de activitate la care puteți participa în mod regulat?

- Activități fizice foarte intense, cum ar fi sărituri sau pivotari, ca în baschet sau fotbal
- Activități fizice intense, cum ar fi muncă fizică intensă, schi sau tenis
- Activități fizice moderate, cum ar fi muncă fizică moderată, alergare sau ‚jogging’
- Activități fizice ușoare, cum ar fi mers pe jos, menaj sau lucru în curte
- Imposibilitatea de a efectua oricare dintre activitățile de mai sus din cauza genunchiului

1. Cum vă afectează genunchiul capacitatea de a:

|  |  | Deloc | Puțin | Moderat | Extrem | Imposibil de efectuat |
| --- | --- | --- | --- | --- | --- | --- |
| a. | Urca scările | **□** | **□** | **□** | **□** | **□** |
| b. | Coborâ scările   \| Coborâ scările \| \| --- \| | **□** | **□** | **□** | **□** | **□** |
| c. | Sta în genunchi | **□** | **□** | **□** | **□** | **□** |
| d. | Face genuflexiuni | **□** | **□** | **□** | **□** | **□** |
| e. | Sta cu genunchiul îndoit | **□** | **□** | **□** | **□** | **□** |
| f. | Ridica de pe scaun | **□** | **□** | **□** | **□** | **□** |
| g- | Alerga drept în față | **□** | **□** | **□** | **□** | **□** |
| h. | Sări și ateriza pe membrul afectat | **□** | **□** | **□** | **□** | **□** |
| i. | Opri și porni repede din loc | **□** | **□** | **□** | **□** | **□** |

**FUNCȚIONALITATE:**

1. Cum ați evalua funcționalitatea genunchiului Dvs pe o scară de la 0 la 10, cu 10 fiind funcționalitate normală, excelenta și 0 fiind incapacitatea de a vă efectua oricare dintre activitățile obișnuite de zi cu zi care pot include sporturi?

FUNCȚIONALITATEA DINAINTEA LEZĂRII GENUNCHIULUI:

Fără limitări în activități obișnuite

# 10

# □

# 9

# □

# 8

# □

# 6

# □

# 7

# □

# 5

# □

# 4

# □

# 3

# □

# 2

# □

# 1

# □

# 0

# □

Nu puteam face activități obișnuite

FUNCȚIONALITATEA ACTUALĂ A GENUNCHIULUI:

# 10

# □

# 7

# □

# 6

# □

# 8

# □

Fără limitări în activități obișnuite

# 9

# □

# 5

# □

# 4

# □

# 3

# □

# 2

# □

# 1

# □

# 0

# □

Nu pot face activități obișnuite
